# Leucocytoclastic vasculitis due to Oxicam

**DOI:** 10.11604/pamj.2017.27.89.11020

**Published:** 2017-06-06

**Authors:** Hafsae Bounniyt, Badredine Hassam

**Affiliations:** 1 University Hospital of Ibn Sina, Department of Dermatology Venerology, Faculty of Medicine and Pharmacy, University Mohammed V Souissi, Rabat, Morocco

**Keywords:** Leukocytoclastic vasculitis, ulcerated nodular lesion, purpuritic lesion, oxicam

## Image in medicine

**Figure 1:**
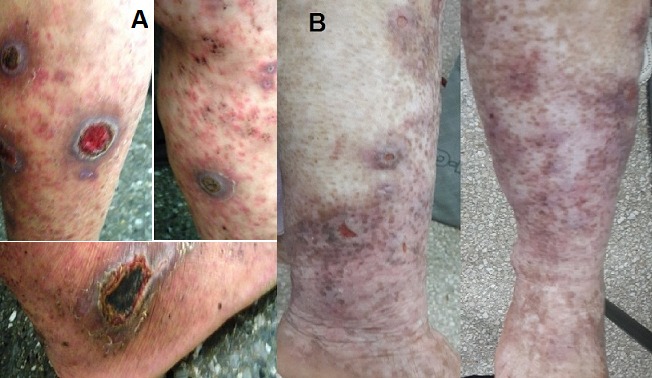
(A) ulcerated nodular lesions with necrotic center; and purpural maculopapular lesions of both lower limbs; (B) evolution after two months of treatment, with persistence of hyper pigmented scars

Vasculitis is an inflammatory disease of blood vessels characterized by the alteration or destruction of the vessel wall. A 55 year’s old woman, followed for diabetes and hypertension, presented to our consultation complaining of generalized pruritic lesions, without fever arthritis or any other systemic manifestations. The patient reported the concept of taking Oxicam; Brexin^®^ for pain of the right wrist and denies any food, drug or environnement allergies. The physical examination showed violaceous petechial purpuric patches, coalescing by area, ulcerated nodular lesions extended to both lower limbs, with presence of necrosis in center of lesions (**Figure 1**A). Histological examination found a perivascular inflammatory cell, and necrosis of the vessels wall. The diagnostic of Leukocytoclastic vasculitis was made. A treatment with colchicine was introduced, with good improvement after two months (**Figure 1**B). Dermatological side effects of Brexin, are fortunately very rare but they should be constantly kept in mind; and the clinician should be aware of uncommon but not rare possibility that a cutaneous eruption could evolve into a significantly more serious reaction.

